# Colistin monotherapy or combination for the treatment of bloodstream infection caused by *Klebsiella pneumoniae*: a systematic review and meta-analysis

**DOI:** 10.1186/s12879-024-09024-6

**Published:** 2024-02-05

**Authors:** Tao Wang, Hongcheng Liu, Huiqing Huang, Yuesong Weng, Xiaojun Wang

**Affiliations:** 1 Center of Clinical Laboratory, The First Affiliated Hospital of Soochow University, 215000 Suzhou, China; 2 Department of Obstetrics and Gynecology, The First Affiliated Hospital of Soochow University, 215000 Suzhou, China; 3 Department of Clinical Laboratory, The Lianyungang Oriental Hospital, 222000 Lianyungang, China; 4 Department of Clinical Laboratory, The Affiliated People’s Hospital of Ningbo University, 315010 Ningbo, China; 5 Department of Clinical Laboratory, Suzhou Wuzhong People’s Hospital, 215100 Suzhou, Jiangsu PR China

**Keywords:** Bloodstream infection of *Klebsiella pneumoniae*, Colistin, Systematic review, meta-analysis

## Abstract

**Background:**

Bloodstream infection of *Klebsiella pneumoniae* (BSI-KP) were associated with increased mortality. *Klebsiella pneumoniae* was tested to susceptible to colistin by E-test and broth microdilution method in clinical laboratory. This study aimed to assess the efficacy of colistin versus tigecycline, carbapenem monotherapy and combination in the treatment of BSI-KP.

**Methods:**

Electronic databases such as PubMed, Web of Science and Embase were searched. The last search was in November 24th, 2022, addressing the colistin, carbapenems and tigecycline monotherapy and combination treatments in patients with BSI-KP. The primary outcomes were 30-day or 28-day mortality. OR where available with 95% CI were pooled in random-effects meta-analysis.

**Results:**

Following the outlined search strategy, a total of 658 articles were identified from the initial database searching. Six studies, 17 comparisons were included. However, they all were observational design, lacking high-quality randomized controlled trials (RCTs). Moderate or low-quality evidences suggested that colistin monotherapy was associated with an OR = 1.35 (95% CI = 0.62–2.97, *P* = 0.45, *Tau*^*2*^ = 0.00, *I*^*2*^ = 0%) compared with tigecycline monotherapy, OR = 0.81 (95% CI = 0.27–2.45, *P* = 0.71, *Tau*^*2*^ = 0.00, *I*^*2*^ = 0%) compared with carbapenem monotherapy. Compared with combination with tigecycline or carbapenem, Colistin monotherapy resulted in OR of 3.07 (95% CI = 1.34–7.04, *P* = 0.008, *Tau*^*2*^ = 0.00, *I*^*2*^ = 0%) and 0.98 (95%CI = 0.29–3.31, *P* = 0.98, *Tau*^*2*^ = 0.00, *I*^*2*^ = 0% ), respectively.

**Conclusions:**

Colistin, carbapenem and tigecycline monotherapy showed similar treatment effects in patients who suffered from BSI-KP. Compared with colistin monotherapy, colistin combined tigecycline therapy might play the synergism effects.

**Trial registration:**

retrospectively registered.

**Supplementary Information:**

The online version contains supplementary material available at 10.1186/s12879-024-09024-6.

## Background

In recent years, *Klebsiella pneumoniae* has evolved into a major clinical and public health threat owing to increasing prevalence of multiple infections caused by producing extended-spectrum β-lactamases (ESBLs) and carbapenemases [1]. The pathogen has long been recognized as an opportunistic pathogen, causing pneumonia, urinary tract infections, and bloodstream infections in immunocompromised individuals [2]. *Klebsiella pneumoniae* was responsible for more than 250,000 deaths associated with antimicrobial resistance, according to global burden of bacterial antimicrobial resistance in 2019 [3]. Clinicians gave great attention to bloodstream infection of *Klebsiella pneumoniae* (BSI-KP), because it caused sepsis shock in patients, which lead to multiple organ dysfunction syndromes (MODS) and death [4]. BSI-KP had a considerable prevalence and high mortality worldwide, leading to the rate between 21 and 69% [5; 6].

According to data from the China Antimicrobial Surveillance Network, *Klebsiella pneumoniae* was resistant to meropenem and imipenem increased to 24.2% and 22.6% in 2022 respectively from 14.1% to 10.3% in 2012 (www.chinets.com). As there are very few new antimicrobials on the market, polymyxins (colistin and polymyxin B) have become an option for treating infections caused by carbapenem-resistant *Klebsiella pneumoniae* (CRKP) [7].

Although *Klebsiella pneumoniae* was proved to susceptible to colistin and tigecycline by E-test and broth microdilution method in clinical laboratory. It remains unknown whether colistin combined therapy can provide a better outcome than colistin monotherapy for the treatment of BSI-KP. In light of these questions, a systematic review and meta-analysis of current evidence was performed to investigate the efficacy of colistin versus tigecycline, colistin versus carbapenem, and colistin combined therapy versus colistin monotherapy for the treatment of BSI-KP.

## Methods

### Search strategies and database selection

We searched PubMed, Web of science and Embase databases. Retrospective, prospective and randomized controlled trials (RCTs) were all included, without any restriction on language. The last search was in November 24th, 2022. The complete search used for PubMed was: (((((((*Klebsiella pneumoniae*[MeSH Terms]) OR (Hyalococcus pneumoniae[Title/Abstract])) OR (Bacterium pneumoniae crouposae[Title/Abstract])) OR (Bacillus pneumoniae[Title/Abstract])) OR (*Klebsiella pneumoniae* aerogenes[Title/Abstract])) OR (Klebsiella rhinoscleromatis[Title/Abstract])) AND (((((((((((((((((Sepsis[MeSH Terms]) OR (Bloodstream Infection[Title/Abstract])) OR (Bloodstream Infections[Title/Abstract])) OR (Infection, Bloodstream[Title/Abstract])) OR (Pyemia[Title/Abstract])) OR (Pyemias[Title/Abstract])) OR (Pyohemia[Title/Abstract])) OR (Pyaemia[Title/Abstract])) OR (Pyaemias[Title/Abstract])) OR (Septicemia[Title/Abstract])) OR (Septicemias[Title/Abstract])) OR (Blood Poisoning[Title/Abstract])) OR (Blood Poisonings[Title/Abstract])) OR (Poisonings, Blood[Title/Abstract])) OR (Poisoning, Blood[Title/Abstract])) OR (Severe Sepsis[Title/Abstract])) OR (Sepsis, Severe[Title/Abstract]))) AND ((((((((Colistin[MeSH Terms]) OR (Polymyxin E[Title/Abstract])) OR (Colimycin[Title/Abstract])) OR (Colisticin[Title/Abstract])) OR (Totazina[Title/Abstract])) OR (Colistin Sulfat[Title/Abstract])) OR (Sulfate, Colistin[Title/Abstract])) OR (Coly-Mycin[Title/Abstract])). If more than one comparison was reported, we included all satisfied comparisons. This systematic review and meta-analysis were reported in accordance with the Preferred Reporting Items for Systematic Reviews and Meta-Analyses (PRISMA) Statement and registration code was CRD42023490911.

### Data extraction and data collection

Two independent investigators (TW and HCL) carried out the initial search, deleted duplicate records, screened the titles and abstracts for relevance, and then identified studies as included, excluded or requiring further assessment. HQH helped resolve any disputes between the two authors. Exclusion criteria included: primarily reviews, meta-analyses, guidelines, editorials, animal studies, no available full-text studies (conference, abstracts), case reports, no extractable data on BSI-KP, in vitro studies, and no reporting primary outcome study.

Considering the low number of RCTs on this subject, no predefined restrictions on study design or study types. The following information were extracted from each study: the first author, length of study, date of publication, country, study design type, bacteria, carbapenemase, carbapenem phenotype, MICs of carbapenem, colistin and tigecycline, setting, number of participants, treatments, clinical outcomes, isolates, type of infection, nephrotoxicity and dosage. The primary outcome was hospital mortality (14-day or 28-day), and secondary outcome included complete microbiological response, or nephrotoxicity.

### Quality assessment

Methodological quality and risk of bias of included studies were determined by the Newcastle–Ottawa Scale (NOS) [8; 9]. The Newcastle–Ottawa Scale assessed the risk of bias in patient selection, comparability between groups, and exposure of outcome, each study was given an eventual score out of a maximum of 9 points. Quality assessment was performed by two authors (TW, HCL), and discrepancies were resolved by YSW.

### Meta-Analysis Approach

Differences were tested as odds ratio (OR) with 95% confidence intervals (CI) for dichotomous outcomes. *I*^*2*^ and *Tau*^*2*^ statistics were used to report heterogeneity. A random-effects model was used for analysis, as we considered clinical heterogeneity between included studies. We assessed the possibility of publication bias by constructing a funnel plot of each trial’s effect size. Besides, considering that the number of articles included were less than 10, we used the Harbord test rather than Egger test to analyze the potential publication bias [10]. The trim-and-fill computation was used to next estimate the effect of publication bias when the publication bias tested [11]. All statistical analyses were performed RevMan software v.5.2 (The Cochrane Collaboration, Copenhagen, Denmark).

## Results

### Study selection

**Fig. 1:**
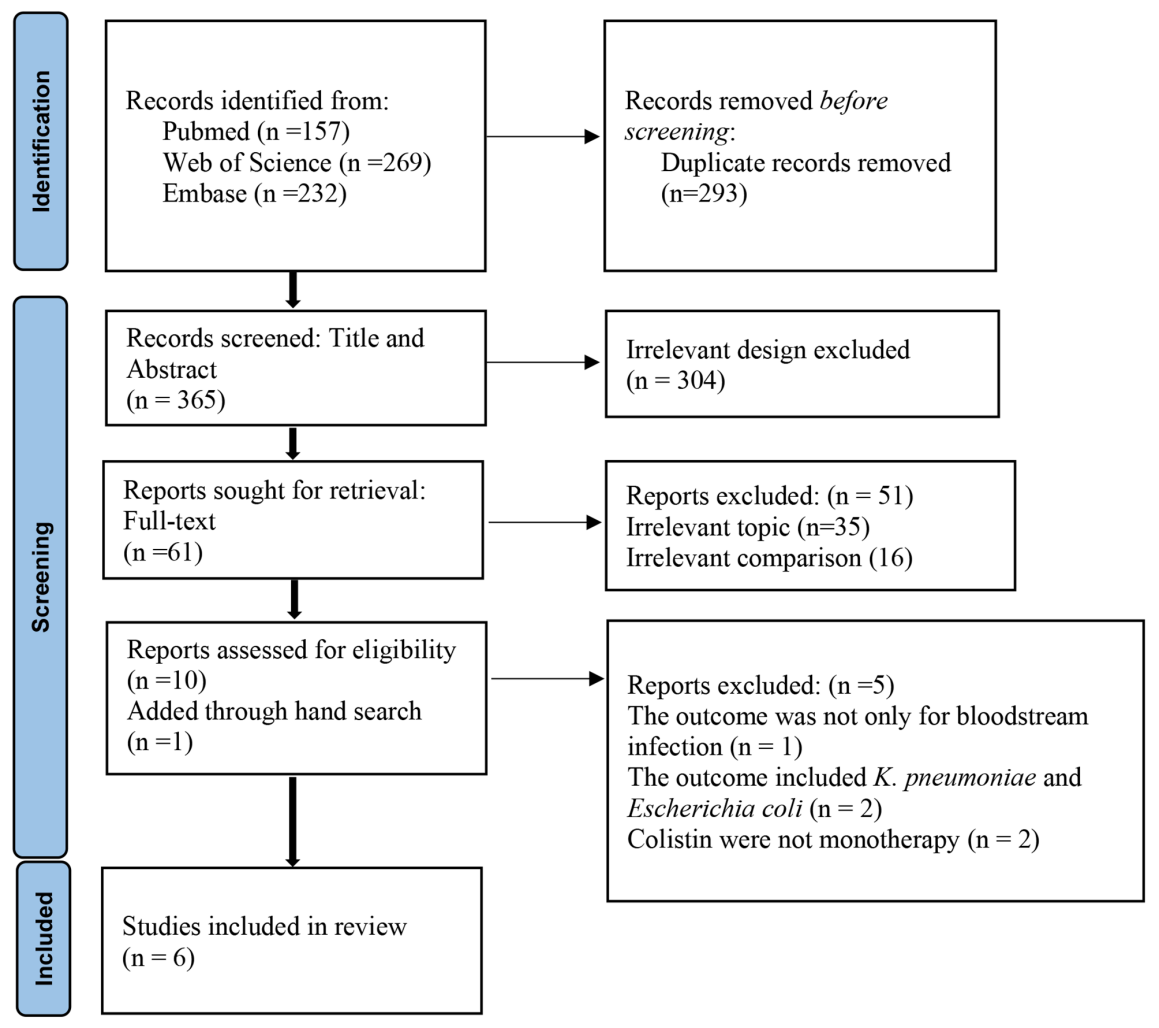
Flow chart of the literature screening process

We included the observational studies to offset the limitations of data analysis because a limited number of RCTs were available. Following the outlined search strategy, a total of 658 articles were identified from the initial database searching, of which 293 records were excluded as duplicates. A total of 304 irrelevant studies were identified by reading the title and/or abstract. After excluding duplicates and irrelevant studies, 61 potentially relevant articles remained. After full-text article review, 51 records were excluded, including 35 irrelevant topic and 16 irrelevant comparisons. The remaining 10 articles were assessed for eligibility, 5 of which were also excluded because one infection type was not only for bloodstream infection, two articles included *K. pneumoniae* and *Escherichia coli*, colistin was not monotherapy in two articles. Further, we included one article through reference hand search. Finally, 6 articles were included in the meta-analysis [12–17]. The flowchart of the literature screening process was showed in Fig. 1.

### Study characteristics

The study characteristics were presented in Table 1. The studies incorporated patients in different hospital setting, times spanned from 2008 to 2019, four countries. Of the 6 included studies, all were retrospective study design and CRKP bloodstream infection. Besides, carbapenemases were detected, *bla*_KPC_ mostly, which expressed resistance to carbapenem. The studies also partly recorded the MICs to colistin and tigecycline. In terms of treatments, three studies compared colistin monotherapy with tigecycline monotherapy [15–17], five studies compared colistin monotherapy with carbapenems monotherapy [12–15; 17], five studies compared colistin monotherapy with colistin combined tigecycline therapy [12; 13; 15–17], four studies compared colistin monotherapy with colistin combined carbapenems therapy [12–15]. Regrettably, only two records reported the dosage of carbapenem, polymyxin and tigecycline [15; 16]. 30-day mortality were collected in four studies [12–14; 16] and 28-day mortality in one study [15]. Since included studies reported total demography features, but different comparison group, these could not be pooled. Data on length of hospital stay, development of resistance and nephrotoxicity were missing, we therefore not report these outcomes. Considering only the RCTs were classified as low risk for selection of participants and confounding, 6 included studies lacked adequate randomization (Table S1 in supplementary).

**Table 1:** Characteristics of included studies

Author	Publication year	Study years	Location	Study type	Bacteria	Carbapenemase	Carbapenem phenotype	Carbapenem MIC (mg/L)	Colistin MIC (mg/L)	Tigecycline MIC (mg/L)	Setting	Infection type	Polymyxin	Time-point	Number of patients	Dosage
Aslan	2022	2014–2018	Turkey	retrospective	KP	*bla*_OXA−48_, *bla*_NDM_, *bla*_VIM_	R	NA	NA	NA	Mix	BSI	colistin	30 day	53	NA
Boszczowski	2019	2010–2013	Brazil	retrospective	KP	*bla*_KPC_	R	NA	> 2	NA	Mix	BSI	colistin	30 day	5	NA
Daikos	2014	2009–2010	Greece	retrospective	KP	*bla*_KPC_, *bla*_VIM_	R	> 8	NA	NA	Mix	BSI	colistin	28 day	89	9 million IU colistin: 2–3 daily 100 to 200 mg tigecycline: 2 daily. 1 g imipenem and 1 g meropenem 3 daily
Papadimitriou-Olivgeris	2021	2010–2019	Greece	retrospective	KP	*bla*_KPC_, *bla*_VIM_, *bla*_NDM_	R	NA	> 2	> 2	ICU	BSI	colistin	30 day	23	NA
Tumbarello	2012	2010–2011	Italy	retrospective	KP	*bla*_KPC_	R	≥ 2	≤ 2	≤ 2	Mix	BSI	colistin	30 day	64	6–9 million IU colistin: 2–3 daily 100-200 mg/day tigecycline. 2 g meropenem 3 daily
Zarkotou	2011	2008–2010	Greece	retrospective	KP	*bla*_KPC_	R	> 1	≤ 2	≤ 2	ICU	BSI	colistin	14 day	22	NA

Abbreviations: KP, *Klebsiella pneumoniae*; NA, not available; BSI, bloodstream infections

### Colistin monotherapy VS Tigecycline monotherapy

**Fig. 2:**
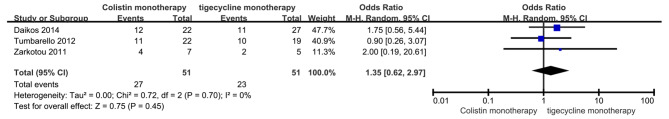
Forest plot of comparison: Colistin monotherapy VS tigecycline monotherapy, outcome: Mortality

**Fig. 3:**
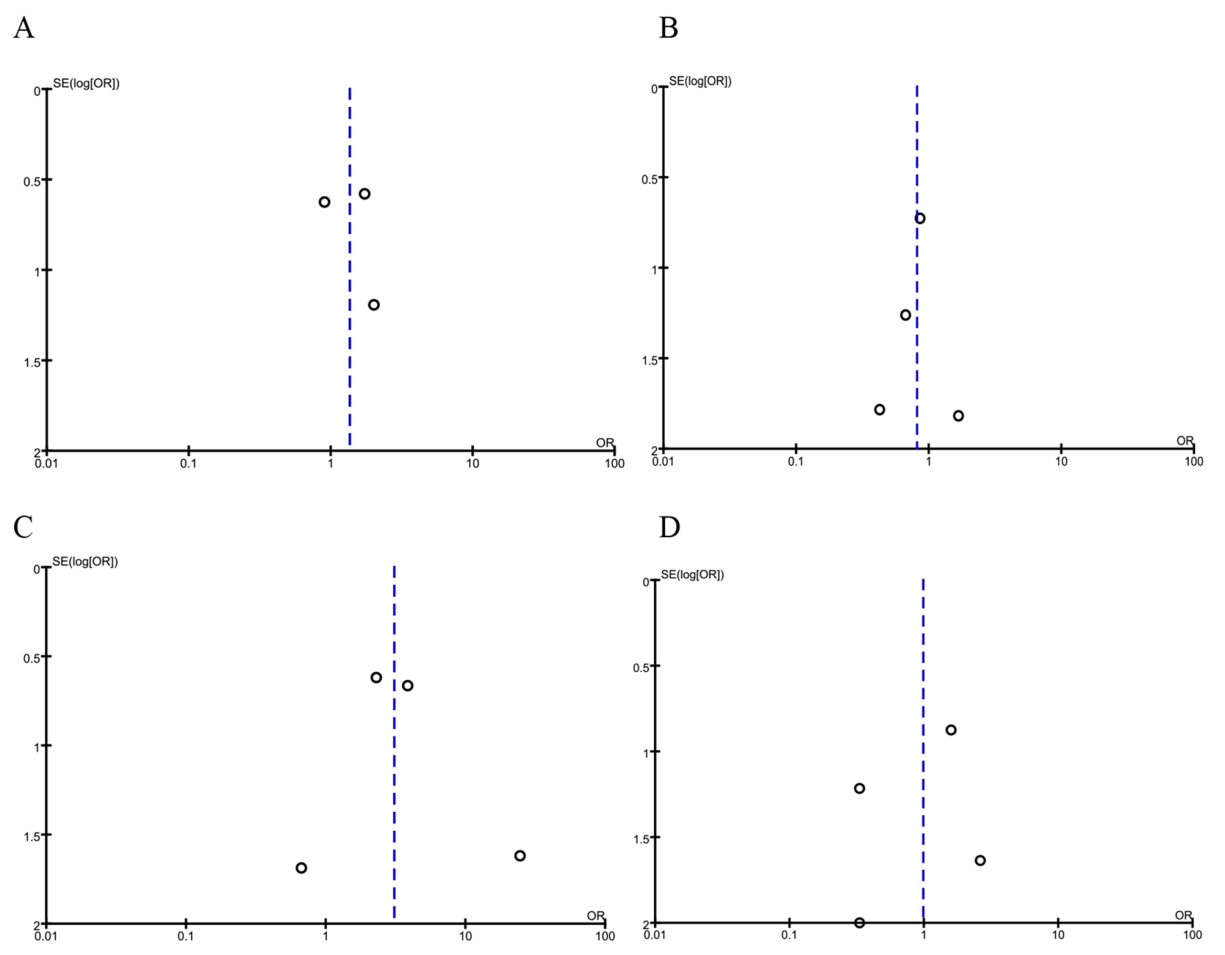
The funnel plots of publication bias of included studies. **A**. Colistin monotherapy VS tigecycline monotherapy. **B**. Colistin monotherapy VS carbapenems monotherapy. **C**. Colistin monotherapy VS combination with tigecycline. **D**. Colistin monotherapy VS combination with carbapenems. outcome: Mortality

As described above, three observational studies reported mortality with colistin monotherapy versus tigecycline monotherapy for the treatment of BSI-KP [15–17]. The clinical cure rate of colistin was comparable with that of tigecycline antibiotics (OR = 1.35, 95% CI = 0.62–2.97, *P* = 0.45, *Tau*^*2*^ = 0.00, *I*^*2*^ = 0%; Fig. 2). The funnel plots of publication bias were shown in Fig. 3A and the publication bias on Harbord test (*P* = 0.833, 95% CI = -22.37-22.40).

### Colistin monotherapy VS Carbapenem monotherapy

**Fig. 4:**
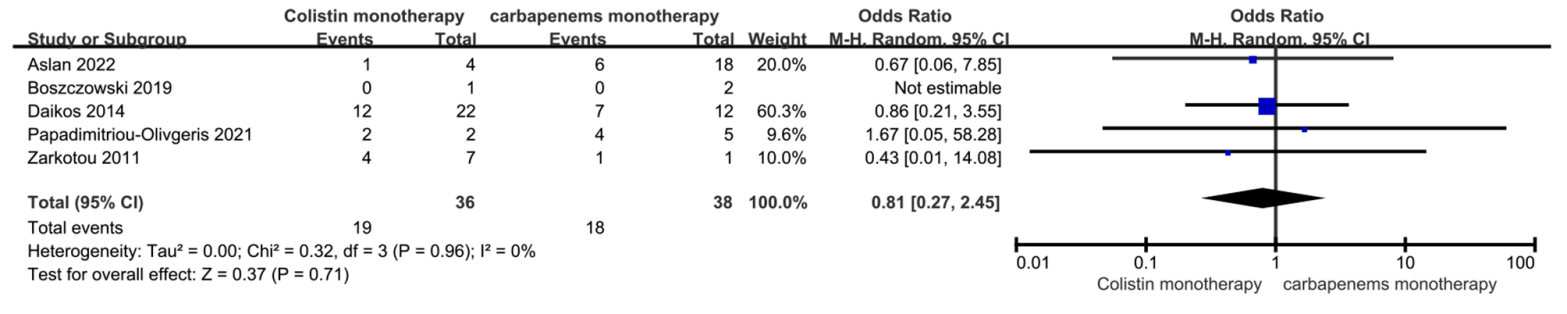
Forest plot of comparison: Colistin monotherapy VS carbapenems monotherapy, outcome: Mortality

Five studies compared colistin monotherapy with carbapenem monotherapy for the treatment of BSI-KP [12–15; 17]. There were no significant differences between the two groups. (OR = 0.81, 95% CI = 0.27–2.45, *P* = 0.71, *Tau*^*2*^ = 0.00, *I*^*2*^ = 0%; Fig. 4). The funnel plots of publication bias were shown in Fig. 3B and the publication bias on Harbord test (*P* = 0.998, 95% CI = -3.53-3.53).

### Colistin monotherapy VS Colistin combined tigecycline therapy

**Fig. 5:**
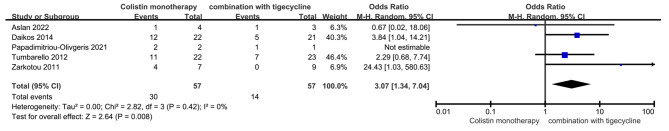
Forest plot of comparison: Colistin monotherapy VS combination with tigecycline, outcome: Mortality

Five studies assessed colistin monotherapy with colistin combined tigecycline therapy for the treatment of BSI-KP [12; 13; 15–17]. Mortality was assessed in a total of 114 patients and produced an OR of 3.07 (95% CI = 1.34–7.04, *P* = 0.008, *Tau*^*2*^ = 0.00, *I*^*2*^ = 0%; Fig. 5). However, included studies lacked high RCTs in favors of the combination therapy. We not find the publication bias on Harbord test (*P* = 0.913, 95% CI = -8.45-7.98), and the funnel plots of publication bias were shown in Fig. 3C.

### Colistin monotherapy VS Colistin combined carbapenems therapy

**Fig. 6:**
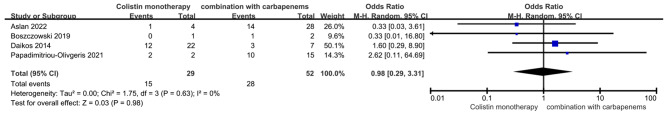
Forest plot of comparison: Colistin monotherapy VS combination with carbapenems, outcome: Mortality

In this analysis, we included combination regimens of colistin with carbapenems. For the analysis of mortality, combinations were examined in 4 studies [12–15]. Together, these studies yieled an OR of 0.98 (95% CI = 0.29–3.31, *P* = 0.98, *Tau*^*2*^ = 0.00, *I*^*2*^ = 0%; Fig. 6). Overall, the quality of the evidence was low. The publication bias on Harbord test (*P* = 0.846, 95% CI = -7.03-7.79) and the funnel plots was shown in Fig. 3D.

## Discussion

We aimed to realize the knowledge to colistin monotherapy versus tigecycline, carbapenem and combination for BSI-KP. This systematic review and meta-analysis of 6 studies suggested that colistin might as effective as carbapenems and colistin combined carbapenems therapy seems not appear to provide better outcomes compared with colistin monotherapy. Colistin showed similar effects as tigecycline and low-quality evidence indicated that colistin combined tigecycline had synergistic acts on BSI-KP.

A previous systematic review and meta-analysis published in 2014 evaluated that the colistin for the treatment of ventilator-associated pneumonia caused by multidrug-resistant Gram-negative bacteria (MDR-GNB-VAP) [8]. It included 11 studies, suggested that colistin was as effective as β-lactam antibiotics for the treatment of MDR GNB VAP and colistin combined therapy also as well as monotherapy. However, *Klebsiella pneumoniae* may possess different feature among GNB. According to a systematic review and meta-analysis by Zusman, 2016, included 7 observational studies for *Klebsiella pneumoniae* bacteremia, polymyxins (polymyxinB, colistin) monotherapy was associated with an OR of 2.09 (95% = 1.21–3.60) for mortality compared with combination therapy with tigecycline [18]. Recently, to assess the effectiveness and safety of colistin among older adults, a study by Margalit included 38 publications (41 comparisons) reported 2857 elderly individuals in 2022, which demonstrated colistin-based therapy resulted in no mortality difference, compared with other regimens, for any infection [19].

Bloodstream infection especially caused by *Enterobacterales*, who received inappropriate empirical treatment approximately 20% in U.S. hospitals, lead to increased risk of mortality [20]. Previous study showed the pooled mortality was much higher than urinary tract infection (UTI; 54.3% versus 13.52%) in patients with carbapenem-resistant *Klebsiella pneumoniae* [21]. Although many studies in the past have shown that colistin was susceptible to *Klebsiella pneumoniae*, the method used for colistin susceptibility testing, microdilution, was just in vitro. Therefore, the systematic review and meta-analysis evaluated the efficacy of colistin in clinic for BSI-KP. To the best of our knowledge, the present research was firstly to investigate the colistin for the treatment of BSI-KP.

Compared with β-lactam-based regimens, colistin increased nephrotoxicity rate by 140% [22].

Therefore, colistin should be regarded as a last-line agent and more cautious alternatives when used. Some studies showed a general message of ‘the more drugs, the better’, a phenomenon that cannot be implemented and maybe unwise. It was worthy to investigate whether MICs of colistin, tigecycline and carbapenem in BSI-KP influenced the treatments option in clinic. However, except for potential side effects, colistin combination therapy might prevent resistance development, getting higher rates of success and shorter treatment periods [23].

Honestly, our study should be interpreted in view of certain limitations. Although we aimed BSI-KP, the present evidences were together from different patient populations, (i.e., KPC, VIM, OXA), and different MICs. Second, all included studies were nonrandomized, retrospective design types. The criteria satisfied our inclusion were just a small number of patients, which may be subject to confounders and bias. Finally, our observational outcome indicators, excluding mortality, were insufficiently detailed, specifically regarding nephrotoxicity and microbial clearance time. Accordingly, these differences might influence the clinical outcomes. Therefore, more well-designed randomized studies of specific patient populations are needed to further clarify this issue.

## Conclusion

*K. pneumoniae* has become a significant problem in terms of public health and clinical outcome and BSI-KP were associated with increased mortality. Electronic databases such as PubMed, Web of Science and Embase were searched. Following the outlined search strategy, a total of 658 articles were identified from the initial database searching. Finally, 6 articles were included in the meta-analysis. We found that colistin, carbapenems and tigecycline were similar treatment effect in patients who suffered from BSI-KP. Compared with colistin monotherapy, colistin combined tigecycline therapy might play the synergism effects due to the low quality of the evidence.

### Electronic supplementary material

Below is the link to the electronic supplementary material.

Supplementary Material 1

## Data Availability

All relevant data are within the manuscript.
