# Supplementary material for: Colistin monotherapy or combination for the treatment of bloodstream infection caused by Klebsiella pneumoniae: a systematic review and meta-analysis
Source: BMC Infect Dis. 2024 Feb 5;24:161. doi: 10.1186/s12879-024-09024-6 (PMC10845734; doi:10.1186/s12879-024-09024-6)
Supplement: Supplementary file 1 — Supplementary Material 1 [file 12879_2024_9024_MOESM1_ESM.docx]

Table S1. Quality of included studies measured by Newcastle-Ottawa Scale.

| Studies | Selection | Comparability | Exposure of outcome | Total |
| --- | --- | --- | --- | --- |
| Aslan, 2022 | ☆ | ☆☆ | ☆☆ | 5 |
| Boszczowski, 2019 | ☆ | ☆ | ☆ | 3 |
| Daikos, 2014 | ☆ | ☆☆ | ☆☆ | 5 |
| Gomez-Simmonds, 2016 | ☆ | ☆☆ | ☆☆ | 5 |
| Nguyen,2010 | ☆ | ☆ | ☆☆ | 4 |
| Papadimitriou-Olivgeris, 2021 | ☆ | ☆☆ | ☆ | 4 |
| Qureshi, 2012 | ☆ | ☆☆ | ☆ | 4 |
| Tumbarello, 2012 | ☆ | ☆☆ | ☆☆ | 5 |
| Zarkotou, 2011 | ☆ | ☆☆ | ☆ | 4 |
